# A new polymorph of white phospho­rus at ambient conditions

**DOI:** 10.1107/S2052252523009247

**Published:** 2023-11-01

**Authors:** Regine Herbst-Irmer, Xiaobai Wang, Laura Haberstock, Ingo Köhne, Rainer Oswald, Jörg Behler, Dietmar Stalke

**Affiliations:** a Institut für Anorganische Chemie, Georg-August-Universität Göttingen, Tammannstraße 4, Göttingen, 37077 Lower Saxony, Germany; b Institut für Physikalische Chemie, Georg-August-Universität Göttingen, Tammannstraße 6, Göttingen, 37077 Lower Saxony, Germany; c Lehrstuhl für Theoretische Chemie II, Ruhr-Universität Bochum, 44780 Bochum, Germany; d Research Center Chemical Sciences and Sustainability, Research Alliance Ruhr, 44780 Bochum, Germany; Formby, Liverpool, United Kingdom

**Keywords:** white phospho­rus, twinning, polymorphs, allotropes

## Abstract

Phospho­rus exists in several different allotropes: white, red, violet and black. For industrial and academic applications, white phospho­rus is the most important. So far, three polymorphs of white phospho­rus, all consisting of P_4_ tetrahedra, have been described. Among these, β-P_4_ crystallizes in the space group *P* 1 and γ-P_4_ in the space group *C*2/*m*. α-P_4_ forms soft plastic crystals with a proposed structure in the cubic space group *I* 43*m* with the lattice constant *a* = 18.51 (3) Å, consisting of 58 rotationally disordered tetrahedra and thus is similar to the structure of α-Mn. Here we present a new polymorph, δ-P_4_. It crystallizes as a sixfold twin with the cell dimensions *a* = 18.302 (2), *b* = 18.302 (2), *c* = 36.441 (3) Å in the space group *P*2_1_2_1_2_1_ with 29 P_4_ tetrahedra in the asymmetric unit. The arrangement resembles the structure of α-Mn, but δ-P_4_ differs from α-P_4_. DFT calculations show δ-P_4_ to be metastable at a similar energy level to that of γ-P_4_.

## Abstract 2

We present δ-P_4_, a new polymorph of white phospho­rus. It crystallizes as a sixfold twin with the cell dimensions *a* = 18.302 (2), *b* = 18.302 (2), *c* = 36.441 (3) Å in the space group *P*2_1_2_1_2_1_ with 29 P_4_ tetrahedra in the asymmetric unit. Although their arrangement resembles the structure of α-Mn as proposed for α-P_4_, δ-P_4_ differs from α-P_4_.

## 1. Introduction

**Figure 1:**
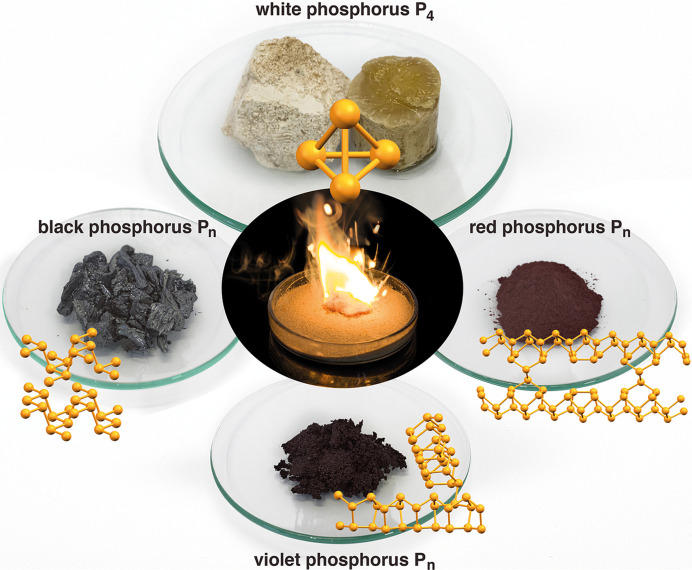
Known modifications of elemental phospho­rus under ambient conditions: their macroscopic manifestations combined with the structures at the atomic level.

Phospho­rus exists in several different modifications: white, red, violet and black (Fig. 1 ▸). Its affinity for oxygen is high, so it exists in nature mainly as phosphates, containing the PO_4_^3–^ anion. This is difficult to activate chemically in contrast to white phospho­rus P_4_, which spontaneously ignites when exposed to air. This makes the element a versatile reagent in organo­phospho­rus chemistry (Iaroshenko, 2019 ▸; Donath *et al.*, 2022 ▸; Grützmacher, 2022 ▸).

The different modifications of phospho­rus vary markedly in their physical properties and chemical reactivity. The most stable modification at room temperature is polymeric black phospho­rus P_*n*_. This polymorph has semiconducting properties. It displays a layered structure consisting of anellated chair-shaped six-membered rings (Fig. 1 ▸, left). At ambient pressure it crystallizes in the space group *Cmca* with *a* = 3.31, *b* = 10.50 and *c* = 4.38 Å (Hultgren *et al.*, 1935 ▸; Brown & Rundqvist, 1965 ▸). Under increasing pressure, it undergoes several phase transitions (Jamieson, 1963 ▸; Kikegawa & Iwasaki, 1983 ▸; Kikegawa *et al.*, 1987 ▸; Scelta *et al.*, 2017 ▸; Marqués *et al.*, 2008 ▸; Akahama *et al.*, 1999 ▸; Sugimoto *et al.*, 2012 ▸); for example, at room temperature the first phase transition at ∼5 GPa leads to the space group *R* 3 *m* with *a* = 3.38 and *c* = 8.81 Å. Fibrous red phospho­rus crystallizes in the space group *P* 1 with *a* = 12.198, *b* = 12.986, *c* = 7.075 Å, and α = 116.99, β = 106.31, γ = 97.91° (Fig. 1 ▸, right) (Ruck *et al.*, 2005 ▸). It consists of tubes with pentagonal cross-sections. The tubes are built from cages of eight or nine phospho­rus atoms connected by dumbbells of two phospho­rus atoms, and interconnect to form a double tube. This is similar to the structure of violet or Hittorf’s phospho­rus, which crystallizes in the space group *P*2/*c* with *a* = 9.21, *b* = 9.15, *c* = 22.60 Å and β = 106.1° (Fig. 1 ▸, bottom) (Thurn & Krebs, 1969 ▸; Zhang *et al.*, 2020 ▸). Zhang *et al.* (2020 ▸) stated that their structure differs slightly from the structure described by Thurn & Krebs (1969 ▸) and discussed the differences [*e.g.* different space groups (*P*2/*n* and *P*2/*c*), *c* axes and ß angles]; however, both structures can be transformed into each other by the matrix −100 0−10 101, hence there is no difference between them but they are identical.

Recently, another structure has been described (Cicirello *et al.*, 2023 ▸) in the space group *C*2/*c* with eleven phospho­rus atoms in the asymmetric unit.^1^ The red and violet P_*n*_ structures differ only in the orientation of the two tubes relative to each other: they are perpendicular to each other in the violet modification and parallel in the red polymorph. Although white phospho­rus is the most toxic and reactive modification, it is a major source for industrial and academic applications (Donath *et al.*, 2022 ▸). To date, three modifications of white phospho­rus have been described in the literature (Simon *et al.*, 1997 ▸). Though the structures for β-P_4_ (Simon *et al.*, 1987 ▸) and γ-P_4_ (Okudera *et al.*, 2005 ▸) are unequivocal, no structural data are available for α-P_4_. γ-P_4_ is the stable low-temperature form and crystallizes in the space group *C*2/*m* with *a* = 9.1709 (5), *b* = 8.3385 (5), *c* = 5.4336 (2) Å and β = 90.311 (3)° with four formula units per cell (*Z* = 4) (Okudera *et al.*, 2005 ▸). It transforms into the intermediate modification β-P_4_, which crystallizes in the space group *P* 1 with *a* = 5.4788 (5), *b* = 10.7862 (11), *c* = 10.9616 (11) Å, α = 94.285 (8), β = 99.653 (7), γ = 100.680 (7)° and *Z* = 6 (Simon *et al.*, 1987 ▸). The room-temperature form α-P_4_ has been described as a plastic crystal (Simon *et al.*, 1997 ▸). As early as 1930, a powder photograph taken at −35°C was interpreted in terms of a cubic cell with *a* = 7.17 Å (Natta & Passerini, 1930 ▸). However, this key structure – ‘a riddle, wrapped in a mystery, inside an enigma’ – remained unsolved. In 1952 single-crystal data were collected in the cubic system with *a* = 18.51 (3) Å, assuming the space group *I* 43*m*, but the structure was never solved, which was attributed to rotational disorder (Corbridge & Lowe, 1952 ▸). As the most reasonable approximation, it was assumed that the centroids of the P_4_ tetrahedra are arranged similar to the atomic positions of the 58 Mn atoms in the complicated structure of α-Mn (von Schnering, 1981 ▸). The most recent verdict is that crystallographic investigations are hampered by a partial transition to red phospho­rus or that a high degree of thermal motion precludes a structure determination (Simon *et al.*, 1997 ▸).

## 2. Experimental

**Figure 2:**
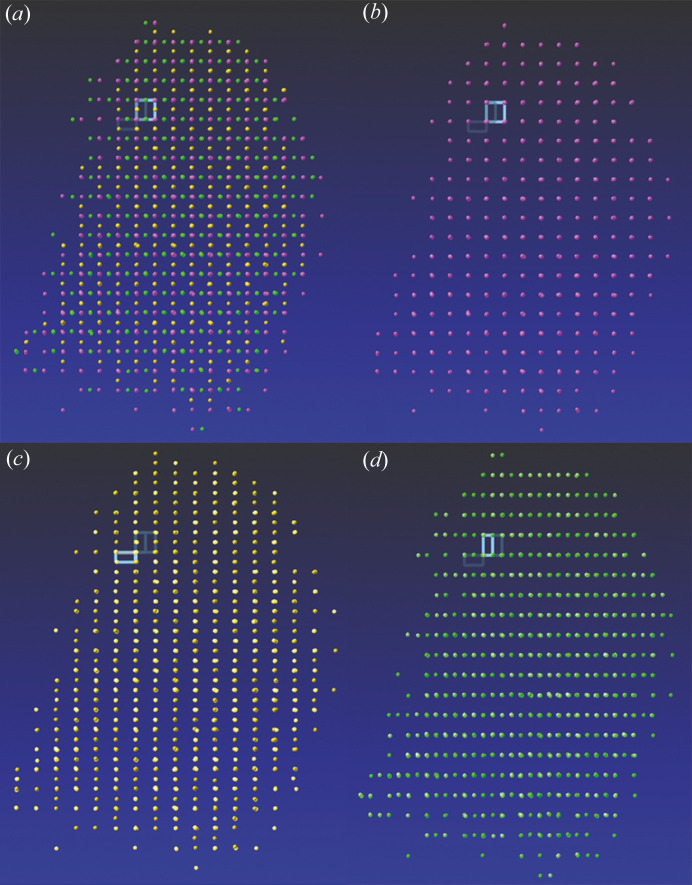
Plot of the Bragg reflections used for indexing (Bruker, 2016 ▸). (*a*) All reflections, (*b*) first, (*c*) second and (*d*) third domain. The light pale rectangles indicate the corresponding cell.

In the course of a synthesis involving the activation of white phospho­rus, we discovered a new polymorph of phospho­rus P_4_, which we introduce in this publication as δ-P_4_. Diffraction data were collected from an apparently cubic crystal with *a* = 36 Å at 100 K. We assumed at first that the data corresponded to the proposed reaction product, not the starting material. The diffraction pattern indicated twinning. Fig. 2 ▸(*a*) shows every second reflection to be missing in every second line. This can be interpreted as a threefold twin, and all reflections can be indexed with three different orientations and cell constants of approximately *a* = 18.3, *b* = 18.3, *c* = 36.5 Å and α = β = γ = 90° [Figs. 2 ▸(*b*)–2 ▸(*d*)]. These cell constants suggest a tetragonal space group. However, the data derived by integration with these three orientation matrices, with subsequent detwinning assuming 4/*m* symmetry, showed only orthorhombic symmetry and hinted at additional twinning that mimicks tetragonal symmetry. This means that there are six twin domains, because each of the three domains has an additional domain, generated by reducing the crystallographic fourfold symmetry to twofold symmetry.

Consequently, the integration was repeated, now using six orientation matrices. The matrices of the twin laws are given in the supporting information. After detwinning, the space group could be determined unequivocally to be *P*2_1_2_1_2_1_. The structure could be solved in this space group (Sheldrick, 2008 ▸) and refined as a sixfold twin without any disorder (Sheldrick, 2015*b* ▸; Sevvana *et al.*, 2019 ▸). The refinement converged to *R*1 [*I* > 2σ(*I*)] = 0.0363. More details can be found in the supporting information.

**Figure 3:**
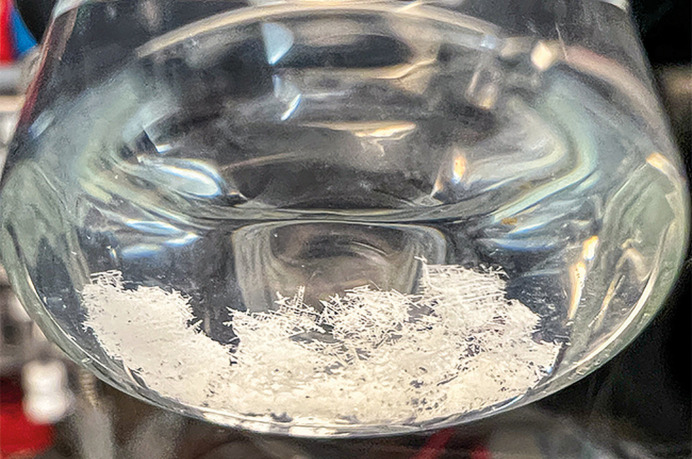
Crystals of the new polymorph.

With these results in hand, we attempted to find suitable crystallization conditions for this new polymorph. The first trials using CS_2_ as the solvent led to soft plastic crystals, as already described in the literature as α-P_4_. They diffracted weakly to a resolution of *ca* 1.6 Å. The cell constants suggested cubic symmetry with *a* = 18.7 Å and the structure could not be solved. Crystallization from dry, degassed hexane gave colourless block-shaped single crystals (see Fig. 3 ▸) which were identified as the δ-polymorph we had just investigated. The crystals were again twinned, and of the same quality as before, leading to the same ordered structure with similar quality indicators (see Table S1 of the supporting information).

## 3. Results

**Figure 4:**
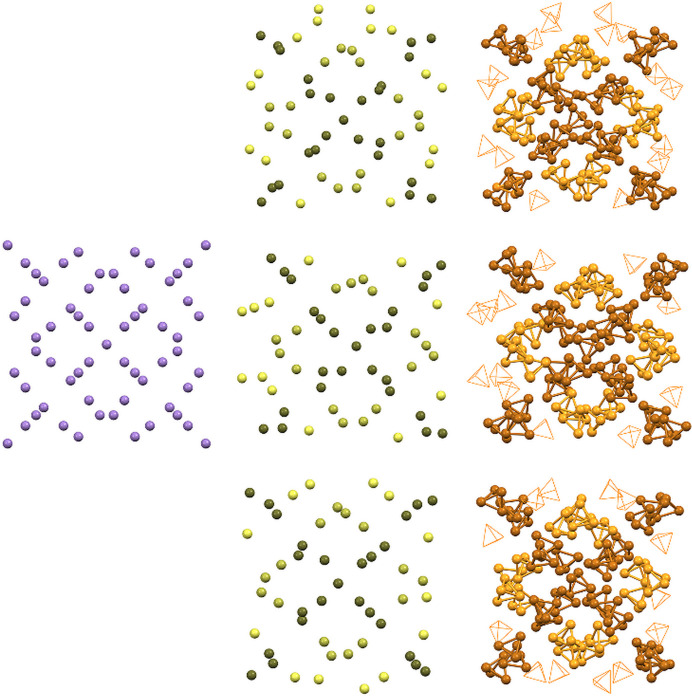
Packing of α-Mn compared with δ-P_4_: left: α-Mn along the *a* axis, which is identical to the *b* and *c* axes caused by the cubic symmetry. Centre: centroids of the P_4_ tetrahedra along the *a* axis (top), *b* axis (middle) and *c* axis (bottom). Right: full tetrahedra of the new δ-P_4_ polymorph along the *a* axis (top), *b* axis (middle) and *c* axis (bottom).

In the literature, the α-P_4_ polymorph is described as a cubic structure with *a* = 18.51 (3) Å in the most probable space group *I* 43*m.* The similarity to α-Mn was mentioned (von Schnering, 1981 ▸). In our δ-P_4_ structure, one axis is doubled and the others are slightly shorter compared with these observations. However, the arrangement of the P_4_ tetrahedra indeed resembles the structure of α-Mn, which crystallizes in space group *I* 43*m* (see Fig. 4 ▸) (Oberteuffer & Ibers, 1970 ▸). The cubic symmetry forces the packing to be identical along all three axes (Fig. 4 ▸, left), whereas they are very similar in the new orthorhombic δ-P_4_ modification (Fig. 4 ▸, right). This can be visualized using the centroids of the δ-P_4_ tetrahedra (Fig. 4 ▸, centre).

**Figure 5:**
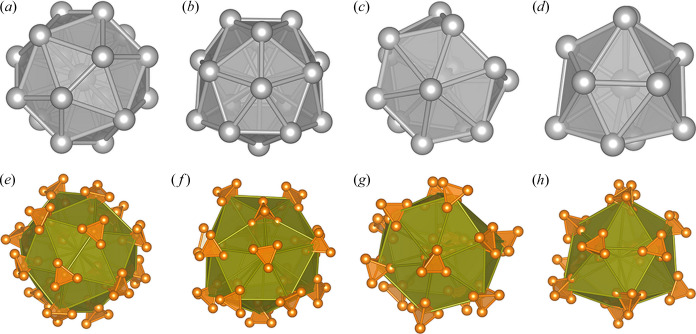
(*a*)–(*d*) Comparison of the α-Mn polyhedra (Momma & Izumi, 2011 ▸) and (*e*)–(*h*) the environment types of the new δ-P_4_ modification. (*a*) α-Mn Type I, (*b*) α-Mn Type II, (*c*) α-Mn Type III and (*d*) α-Mn Type IV, (*e*) δ-P_4_ Type I, (*f*) δ-P_4_ Type II, (*g*) δ-P_4_ Type III and (*h*) δ-P_4_ Type IV.

The asymmetric unit consists of 29 P_4_ tetrahedra. A *Z*′ value as high as 29 might lead one to think of modulation. However, the typical signs of modulation (strong central reflections with weaker satellites) were absent from the diffraction pattern. Additionally, we note the similarity to the non-modulated structure of α-Mn with similar environments of the Mn atoms and the P_4_ tetrahedra. In α-Mn there are four independent Mn atoms with different crystallographic symmetry and environments (Types I–IV) corresponding to 58 atoms in the unit cell. Two belong to Type I [Fig. 5 ▸(*a*)], eight to Type II [Fig. 5 ▸(*b*)], 24 to Type III [Fig. 5 ▸(*c*)] and 24 to Type IV [Fig. 5 ▸(*d*)]. Similar environments can be observed for the P_4_ units of the δ-P_4_ structure: thus there is one tetrahedron of Type I [Fig. 5 ▸(*e*)], four of Type II [Fig. 5 ▸(*f*)], 12 of Type III [Fig. 5 ▸(*g*)] and 12 of Type IV [Fig. 5 ▸(*h*)], exactly half as many of each type as for α-Mn, coordinated in the same way as in α-Mn (see Figs. 5 ▸ and 6 ▸ and the supporting information). Therefore, our new δ-P_4_ polymorph indeed resembles α-Mn, as commented by von Schnering (1981 ▸), but it is definitely different from the α-P_4_ form discussed in previous publications (Simon *et al.*, 1997 ▸, 1987 ▸; Corbridge & Lowe, 1952 ▸).

**Figure 6:**
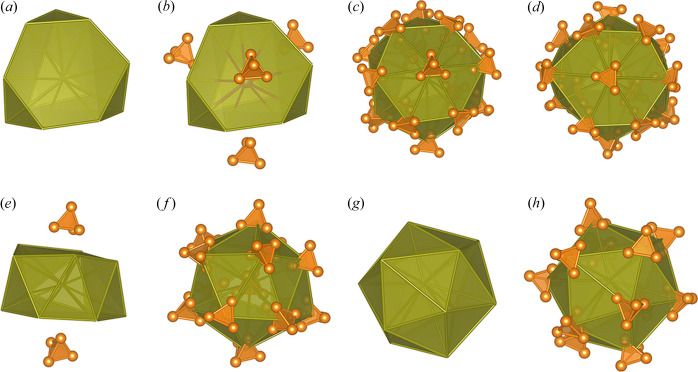
Polyhedra of the four environment types: (*a*) truncated tetrahedron, (*b*) truncated tetrahedron with the hexagonal planes capped by P_4_ tetrahedra, (*c*) all P_4_ tetrahedra of Type I, (*d*) all tetrahedra of Type II, (*e*) hexagonal antiprism with one edge missing of Type III and the hexagonal planes capped by P_4_ tetrahedra, (*f*) all P_4_ tetrahedra of Type III, (*g*) icosahedron, (*h*) icosahedron of Type IV with all P_4_ tetrahedra (Momma & Izumi, 2011 ▸).

The P_4_ tetrahedra of Types I [Fig. 6 ▸(*c*)] and II [Fig. 6 ▸(*d*)] are surrounded by 16 further tetrahedra, and the resulting global polyhedron can be described as a distorted and truncated tetrahedron [Fig. 6 ▸(*a*)] with 4 tetrahedra above the sixfold planes [Fig. 6 ▸(*b*)]. The distortion is higher for Type II. The tetrahedra of Type IV [Fig. 6 ▸(*h*)] are coordinated by 12 further tetrahedra, leading to a distorted icosahedron [Fig. 6 ▸(*g*)], whereas the polyhedron of the Type III tetrahedra [Fig. 6 ▸(*f*)], with coordination number 13, can be described as a hexagonal antiprism with one corner missing and capped by two tetrahedra above and below [Fig. 6 ▸(*e*)].

Our new polymorph δ-P_4_ and the old α-P_4_ are correlated in the following way: in both the P_4_-tetrahedra probably show an overall structural pattern reminiscent of α-Mn, provided that only the centroids of the tetrahedra are taken into account. Compared with α-P_4_, in the new δ-P_4_ polymorph all 29 tetrahedra in the asymmetric unit are ordered (rather than disordered), one cell axis is doubled, the cell is primitive and not *I*-centred, and the symmetry is reduced from cubic to orthorhombic. None of the tetrahedra are related by translational or rotational symmetry. In α-Mn and presumably also in α-P_4_ there are only 29/24 atoms or tetrahedra, respectively, in the asymmetric unit. Therefore, severe rotational P_4_-disorder is introduced, permitting only low-resolution diffraction data for α-P_4_. The reduction of symmetry in δ-P_4_ is accompanied by twinning. A further distinction can be found in the densities, with a value of 1.96 g cm^–3^ for δ-P_4_ at 100 K but only 1.83 g cm^−3^ at 100 K in α-P_4_, based on the data we collected from the soft plastic crystals (see above), assuming 58 tetrahedra in a unit cell.

With the new δ-P_4_ polymorph stable at ambient conditions, its energetic relationships to the other known polymorphs remained to be determined. Accordingly, the relative stabilities of the different polymorphs of phospho­rus have been computed by density-functional theory (DFT) methods, employing the generalized gradient approximation of Perdew, Burke and Ernzerhof (PBE) (Perdew *et al.*, 1996 ▸, 1997 ▸). Since dispersion corrections are known to be important for elemental phospho­rus (Aykol *et al.*, 2017 ▸), not only uncorrected PBE calculations but also D3 and D3/BJ dispersion corrections, as developed by Grimme and coworkers, have been performed (Grimme, 2006 ▸; Grimme *et al.*, 2010 ▸, 2011 ▸).

Taking black phospho­rus as the reference, the relative binding energies (Table S2) show that the correct order of stability can only be achieved by including dispersion corrections, in agreement with previous work (Aykol *et al.*, 2017 ▸). Further, the energetic ordering of γ-P_4_ and δ-P_4_ changes from PBE to PBE-D3, but the absolute value of the energy difference is very small. Becke–Johnson (BJ) damping increases the energy difference significantly. Consistent with previous theoretical studies (Aykol *et al.*, 2017 ▸; Bachhuber *et al.*, 2014 ▸) on a number of the polymorphs, the D3 and D3/BJ values show that the individual P_4_ moieties are held together by van der Waals forces, resulting in metastable γ-P_4_ and δ-P_4_ polymorphs.

## 4. Conclusions

We have determined the crystal structure of a new polymorph of white phospho­rus which resembles the structure of α-Mn. This similarity has already been described for α-P_4_ (von Schnering, 1981 ▸). However, many other aspects of α-P_4_ do not apply to our crystals of the new δ-P_4_ polymorph. While the former crystallizes from CS_2_, the latter crystallizes from dry, degassed hexane. The structure refinement of cubic α-P_4_ is precluded by rotational disorder, but the orthorhombic δ-P_4_ polymorph was refined without disorder despite serious twinning. Additionally, the two forms differ in their densities. From DFT calculations it can be concluded that γ-P_4_ and δ-P_4_ are metastable and that the twinning of δ-P_4_ is energetically favourable. The description of the new polymorph significantly extends our knowledge of phospho­rus.

## 5. Related literature

The following references are cited in the supporting information: Belsky *et al.* (2002 ▸); Bruker (2021*a* ▸,*b* ▸); Herbst-Irmer (2016 ▸); Kottke & Stalke (1993 ▸); Kresse & Furthmüller (1996 ▸); Kresse & Joubert (1999 ▸); Schulz *et al.* (2009 ▸); Sheldrick (2015*a* ▸,*c* ▸).

## Supplementary Material

Crystal structure: contains datablock(s) twin5, twin5_2. DOI: 10.1107/S2052252523009247/lt5062sup1.cif

Structure factors: contains datablock(s) twin5. DOI: 10.1107/S2052252523009247/lt5062twin5sup2.hkl

Structure factors: contains datablock(s) twin5_2. DOI: 10.1107/S2052252523009247/lt5062twin5_2sup3.hkl

Supporting figures and tables. DOI: 10.1107/S2052252523009247/lt5062sup4.pdf

CCDC references: 2268211, 2268212

## Footnotes

1 The cell parameters are nearly identical to the structure refined in *P*2/*n* with P_21_ in the asymmetric unit by Zhang *et al.* (2020 ▸) and hence to Thurn & Krebs (1969 ▸). The data quality of Cicirello *et al.* (2023 ▸) is very poor. In the Checkcif report there are several A alerts, *e.g.* no anisotropic refinement, max residual density of 4.65 e Å^−3^, *wR*2 = 0.49. It is possible to refine the coordinates of the structure deposited by Zhang *et al.* (2020 ▸) against the data deposited by Cicirello *et al.* (2023 ▸). Of course, half of the data are then missing and 5 of the 21 P atoms adopt negative *U*_iso_ values. However, *wR*2 reaches 0.29 and the highest residual density is 2.09 e Å^−3^. Therefore, there is reasonable doubt whether this is really a new structure and whether P_11_ is indeed the content of the asymmetric unit, rather than P_21_ as in the original structure by Thurn & Krebs (1969 ▸).
