# Supplementary material for: A new polymorph of white phospho­rus at ambient conditions
Source: IUCrJ. 2023 Nov 1;10(Pt 6):766–71. doi: 10.1107/S2052252523009247 (PMC10619451; doi:10.1107/S2052252523009247)
Supplement: Supplementary file 4 [file m-10-00766-sup4.pdf]

IUCrJ

Volume 11 (2024)

Supporting information for article:

A New Polymorph of White Phosphorus at Ambient Conditions

Regine Herbst-Irmer, Xiaobai Wang, Laura Haberstock, Ingo Köhne, Rainer Oswald, Jörg Behler and Dietmar Stalke

S1. Crystallization

White phosphorus, kept under water, was cut into pieces of 1.5 to 2 g, then quickly removed from water and immersed in dry hexane (50 mL). A single lump was cleaned using an ultrasonic bath and the solid was then quickly transferred with tweezers into another batch of dry hexane. This step needs to be repeated at least three times. Finally, the solid material was transferred to an argon-protected Schlenk flask. Under Schlenk conditions, 10 mL dry and degassed hexane was added. Stirring and heating the mixture to 50°C yielded a clear solution, which was nevertheless carefully filtered. The crystal suitable for single crystal structure determination was obtained from the filtrate at 0°C after 3 d. The repeated steps of washing the white phosphorus are vital because water seriously affects the crystallization. If small water droplets remain in the Schlenk flask before the final filtration step, this material should under no circumstances be added to the final filtrate. Although a very small amount of water will remain dissolved in the hexane (solubility 9.5 mg/L at 25°C), this is negligible because it does not seem to affect the crystallization.

S2. Crystal Structure Determination

S2.1. Data Collection

The crystals, coated with a drop of inert oil, were transferred to the diffractometer at low temperature using the X-Temp2 device (Kottke & Stalke, 1993). The data were collected at 100 K with Mo $K\alpha$ radiation on a Bruker SMART APEX II system based on D8 three-circle goniometers with Incoatec microfocus X-ray sources (IµS) and Incoatec QUAZAR mirror optics (Schulz *et al.*, 2009). Data were collected for two crystals. For crystals 1 and 2, two or nine 0.5° omega scans were performed, respectively.

S2.2. Cell Determination

The automatic procedure in *APEX* (Bruker Nano Inc., 2021a) found a cubic cell with a = 36.5 Å. However, the diffraction pattern clearly indicated twinning. This can be interpreted as a three-fold twinning, and all reflections can be indexed with three different orientations and cell constants of approximately a = 18.3, b = 18.3, c = 36.5 Å and $\alpha = \beta = \gamma = 90$ °.

S2.3. Integration and Space Group Determination

The cell constants suggest a tetragonal space group. The integration was performed with *SAINT* (Bruker Nano Inc., 2021b) with three orientation matrices. *TWINABS* (Sevvana *et al.*, 2019) was then used to detwin the data using Laue group 4/*m*. The fractional contributions of the three domains were determined to be 0.17, 0.45 and 0.38. The R_{int} was 0.1563 for all reflections of all domains. *XPREP* (Sheldrick, 2015c) could not find an acceptable space group consistent with the systematic absences.

The mean value for $|E^2\text{-}1|$ was 0.657, the low value being a warning sign of additional pseudo-merohedral twinning (Herbst-Irmer, 2016). Therefore, *TWINABS* was repeated assuming Laue group *mmm*. Now the fractional contributions were determined to be 0.13, 0.47 and 0.40, while R_{int} improved to 0.086. Again the systematic absences were not quite clear but could be interpreted as consistent with space group $P2_12_12_1$.

Systematic absence exceptions:

	b--	c--	n--	**21--**	-c-	-a-	-n-	-21-	--a	--b	--n	--21
N	334	369	341	**7**	347	333	342	**5**	155	155	160	**14**
N I>3σ	252	290	250	**3**	273	246	237	**0**	81	81	80	**0**
<I>	27.4	21.7	21.7	**8.2**	24.0	26.4	22.2	**1.3**	15.6	15.6	10.2	**0.6**
<I/σ>	14.2	15.9	13.1	**4.4**	15.9	12.9	11.7	**1.7**	5.0	5.3	5.1	**1.5**

The mean value for $|E^2\text{-}1|$ was 0.614 even lower than before. Neither the program *SHELXT* (Sheldrick, 2015a) nor *SHELXD* (Sheldrick, 2008) could solve the structure. Additional pseudo-merohedral twinning was taken into account and the integration was repeated, now with six orientation matrices (see Figure S1). *TWINABS* determined the fractional contributions to be 0.39, 0.35, 0.04, 0.08, 0.04, and 0.08, with a corresponding R_{int} of 0.1000. An R_{int} value of 0.516 for the tetragonal Laue group 4/*m* proved the tetragonal symmetry to be wrong. *XPREP* found the systematic absences for $P2_12_12_1$ and $|E^2\text{-}1|$ was 0.714.

Systematic absence exceptions:

	b--	c--	n--	**21--**	-c-	-a-	-n-	**-21-**	--a	--b	--n	**--21**
N	375	382	383	**7**	381	372	381	**8**	190	189	195	**16**
N I>3σ	196	239	201	**0**	240	183	193	**0**	76	78	68	**0**
<I>	24.9	23.8	21.2	**0.9**	19.0	21.3	16.6	**1.6**	14.8	15.1	9.1	**0.6**
<I/ σ >	5.3	6.6	4.9	**0.2**	6.7	5.2	5.0	**0.5**	2.9	2.9	2.5	**0.8**

domain1 → domain2
$$\begin{pmatrix} -1 & 0 & 0 \\ 0 & 0 & -0.5 \\ 0 & -2 & 0 \end{pmatrix}$$

domain1 → domain3
$$\begin{pmatrix} 0 & 0 & -0.5 \\ 0 & -1 & 0 \\ -2 & 0 & 0 \end{pmatrix}$$

domain1 → domain4
$$\begin{pmatrix} 0 & 1 & 0 \\ 1 & 0 & 0 \\ 0 & 0 & -1 \end{pmatrix}$$

domain1 → domain5
$$\begin{pmatrix} 0 & 0 & -0.5 \\ -1 & 0 & 0 \\ 0 & 2 & 0 \end{pmatrix}$$

domain1 → domain6
$$\begin{pmatrix} 0 & -1 & 0 \\ 0 & 0 & -0.5 \\ 2 & 0 & 0 \end{pmatrix}$$

domain2 → domain3
$$\begin{pmatrix} 0 & 1 & 0 \\ 0 & 0 & 0.5 \\ 2 & 0 & 0 \end{pmatrix}$$

domain2 → domain4
$$\begin{pmatrix} 0 & 0 & -0.5 \\ -1 & 0 & 0 \\ 0 & 2 & 0 \end{pmatrix}$$

domain2 → domain5
$$\begin{pmatrix} 0 & 1 & 0 \\ 1 & 0 & 0 \\ 0 & 0 & -1 \end{pmatrix}$$

domain2 → domain6
$$\begin{pmatrix} 0 & 0 & 0.5 \\ 0 & 1 & 0 \\ -2 & 0 & 0 \end{pmatrix}$$

domain3 → domain4
$$\begin{pmatrix} 0 & -1 & 0 \\ 0 & 0 & -0.5 \\ 2 & 0 & 0 \end{pmatrix}$$

domain3 → domain5
$$\begin{pmatrix} 1 & 0 & 0 \\ 0 & 0 & 0.5 \\ 0 & -2 & 0 \end{pmatrix}$$

domain3 → domain6
$$\begin{pmatrix} 0 & 1 & 0 \\ 1 & 0 & 0 \\ 0 & 0 & -1 \end{pmatrix}$$

domain4 → domain5
$$\begin{pmatrix} 0 & 0 & 0.5 \\ 0 & -1 & 0 \\ 2 & 0 & 0 \end{pmatrix}$$

domain4 → domain6
$$\begin{pmatrix} -1 & 0 & 0 \\ 0 & 0 & 0.5 \\ 0 & 2 & 0 \end{pmatrix}$$

domain5 → domain6
$$\begin{pmatrix} 0 & 0 & -0.5 \\ 1 & 0 & 0 \\ 0 & -2 & 0 \end{pmatrix}$$

Figure S1 Twin matrices

S2.4. Structure Solution and Refinement

The structure was solved in $P2_12_12_1$ with *SHELXD* (Sheldrick, 2008); 116 phosphorous atoms in 29 P_4 tetrahedra were found. The refinement with *SHELXL* (Sheldrick, 2015b) was then performed against the twinned data using only the reflections with contribution of the main component (Table S1). No disorder was observed.

Table S1 Crystallographic data

Crystal	1	2
CCDC	2268211	2268212
Empirical formula	P_4	P_4
Mol. w. (g mol^{-1})	123.88	123.88
Temperature (K)	100(2)	100(2)
Wavelength (Å)	0.71073	0.71073
Crystal system	orthorhombic	orthorhombic
Space group	$P2_12_12_1$	$P2_12_12_1$
a (Å)	18.302(2)	18.298(2)
b (Å)	18.302(2)	18.298(2)
c (Å)	36.441(3)	36.408(3)
V (Å^3)	12206(2)	12190(2)
Z	116	116
Density (Mg/m^3)	1.955	1.958
Max. θ. (°)	25.897	26.124
Refl. measured	52805	340118
Refl. unique	12639	13397
R_{int}	0.0723	0.0974
Data / restraints / parameters	12639 / 0 / 1050	13397 / 0 / 1050
$R1$ ($I > 2\sigma(I)$)	0.0363	0.0338
$wR2$ (all data)	0.0836	0.0768
Largest diff. peak and hole (eÅ^{-3})	0.476 / -0.435	0.660 / -0.415
Fractional contributions: Domain 1	0.3461	0.3133
Domain 2	0.3247(10)	0.1473(8)
Domain 3	0.0528(8)	0.1174(8)
Domain 4	0.1113(4)	0.1175(4)
Domain 5	0.0768(8)	0.0981(8)
Domain 6	0.0883(8)	0.2064(9)

S3. Structural Information

The 29 tetrahedra in the asymmetric unit are closely similar with P-P bond lengths in the range 2.138(5)–2.195(5) Å, with a mean value of 2.174 Å. The P-P-P bond angles lie in the range 59.03(14)–61.21(14)°, with a mean value of 60.0°.

S3.1. Details of the Structure of α-Mn compared to δ-P_4

Mn1 occupies Wyckhoff position 2a (Type I) with $\bar{4}3m$ symmetry, Mn2 position 8c (Type II) with $3m$ symmetry, Mn3 (Type III) and Mn4 (Type IV) position 24g with m symmetry. Mn1 is coordinated by 4 atoms of Type II and 12 of Type IV, Mn2 by one atom of Type I, 6 of Type III and 9 of Type IV, Mn3 by 2 atoms of Type II, 6 of Type III and 5 of Type IV, and Mn4 by one of Type I, 3 of Type II, 5 of Type III and 3 of Type IV. In the original paper (Oberteuffer & Ibers, 1970), there is a typing error in Table 5, where the coordination of Mn3 is described with one coordination to an atom of Type II and seven to Type III. However, the coordination with the distance of 2.930 Å is to Mn2 and not to Mn3.

The same structural pattern holds for the P_4 tetrahedra in δ-P_4: One tetrahedron of Type I is surrounded by 4 tetrahedra of Type II and 12 of Type IV, 4 tetrahedra of Type II are surrounded by one tetrahedron of Type I, 6 of Type III and 9 of Type IV, 12 tetrahedra of Type III are surrounded by 2 tetrahedra of Type II, 6 of Type III and 5 of Type IV, and 12 tetrahedra of Type IV are surrounded by one of Type I, 3 of Type II, 5 of Type III and 3 of Type IV.

S4. Theoretical Investigation

For all calculations, version 6.3.1 of the Vienna *ab initio* simulation package (*VASP*) (Kresse & Furthmüller, 1996; Kresse & Joubert, 1999) was used. Gamma-centered k-point grids of size 15x5x11 for black phosphorus, 4x5x9 for red phosphorus, 5x5x2 for violet phosphorus, 5x6x9 for γ-P_4, and 3x3x1 for δ-P_4 were employed. The convergence criteria for the energies and the forces were set to 10^{-6} eV and $1.5*10^{-3}$ eV/Å, respectively.

Table S2 Calculated relative binding energies

polymorph/ΔE[meV/atom]	PBE	PBE-D3	PBE-D3/BJ
black	0	0	0
red	-24.95	-0.91	22.38
violet	-26.45	-3.79	24.07
γ-P_4	121.63	158.13	227.55
δ-P_4	119.65	160.71	239.06

The crystal structures were fully relaxed with respect to the lattice parameters and atomic positions, starting from the experimental values, which were taken from the Inorganic Crystal Structure Database (ICSD) (Belsky *et al.*, 2002) for the known allotropes and taken from experiments reported here for δ-P_4. The lattice relaxations were performed for the primitive cells for all structures, and we note that, in

the case of δ-P_4, the primitive cell of the detwinned structure experimentally includes 116 P_4 tetrahedra, resulting in a total of 464 atoms.

The analysis of the calculated lattice parameters given in Table S3 shows an increasing deviation from the experimental values over all the allotropes, with the largest change for δ-P_4 when dispersion is taken into account. The overbinding tendency of PBE-D3/BJ is large for all systems investigated here.

Table S3 Lattice parameters of the primitive cells

	black	red	violet	γ-P_4	δ-P_4
experiment					
a	3.3136(5)	12.198(8)	9.210(2)	9.1709(5)	18.302(2)
b	10.478(1)	12.986(8)	9.128(2)	8.3385(5)	18.302(2)
c	4.3763(5)	7.075(7)	21.893(6)	5.4336(2)	36.441(3)
PBE					
a	3.3094	12.5949	9.2704	9.4448	18.7675
b	10.8749	13.0242	9.2056	8.4565	18.7236
c	4.4779	7.2992	22.7083	5.5849	37.2413
PBE-D3					
a	3.3073	12.0731	9.2150	9.0773	18.2089
b	10.5143	12.9962	9.0999	8.0758	18.1365
c	4.3727	7.0255	21.8253	5.4634	36.1294
PBE-D3/BJ					
a	3.3238	11.4946	9.1377	8.7797	17.4938
b	10.1108	12.9352	8.9879	7.7902	17.4383
c	4.1179	6.7850	20.9066	5.2621	34.6348

This can be explained partially by the size of the cell used for calculation, as can be seen from values given in Table S4, which show a strong variation of the lattice parameters with the size of the cell used because of the reduction of the symmetry. The resulting stacking along the **a**-axis is visualized in Figure S4. For the PBE calculations, the difference between a and b decreases from 0.0439 Å to 0.0265 Å. Stacking along the **c**-axis (1x1x2) results in changes of the same magnitude. Taking dispersion into consideration, the effects are less pronounced but lead to an elongation of the lattice parameters.

Table S4 PBE and PBE-D3 δ-P_4 lattice parameters for different supercells normalized to the primitive cell

	PBE			PBE-D3		
Lattice parameter/supercell	(1x1x1)	(2x1x1)	(1x1x2)	(1x1x1)	(2x1x1)	(1x1x2)
a	18.7675	18.7218	18.7255	18.2089	18.2167	18.2181
b	18.7236	18.6954	18.6982	18.1365	18.1530	18.1528
c	37.2413	37.1767	37.1710	36.1294	36.1710	36.1688

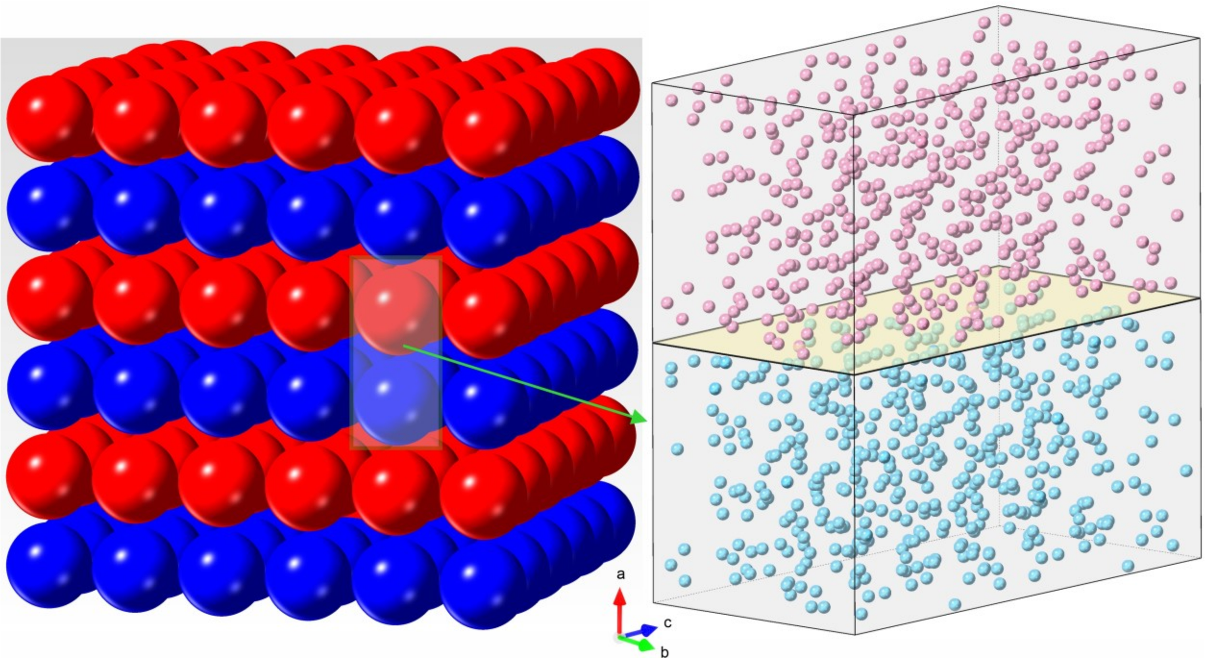

Figure S2 Stacking along the a-axis with red and blue spheres representing the primitive cells. The right-hand panel shows the (2x1x1) supercell containing 928 P atoms.

S5. Hazards

White phosphorus is extremely pyrophoric when exposed to air. At all stages it should be handled under protective conditions, i.e. under water, argon or nitrogen gas. In addition, white phosphorus is toxic (LD_{50} (oral, rate): 3.0 mg/kg; maximum workplace concentration: 0.01 mg/m^3). Contaminated areas should immediately be treated with aqueous $CuSO_4$-solutions to give the insoluble and non-bioavailable Cu_3P_2.
